# Proceedings from The Consortium for Technology & Innovation in Pediatrics (CTIP) 2024 Annual Pediatric Device Innovation Symposium

**DOI:** 10.1186/s12919-025-00315-7

**Published:** 2025-03-24

**Authors:** Tamara P. Lambert, Grzegorz Zapotoczny, Bianca Riello, Nadine Afari, Yaniv Bar-Cohen, Madison Christmas, Salima Jamal, Shahida Qazi, Melissa A. Bent, Juan Espinoza

**Affiliations:** 1 Ann & Robert H. Lurie Children’s Hospital of Chicago, Chicago, IL USA; 2 Feinberg School of Medicine, Northwestern University, Chicago, IL USA; 3 Children’s Hospital Los Angeles, Los Angeles, CA USA

**Keywords:** Medical device development, Pediatrics, Innovation, Proceedings, Pediatric medical devices, United States Food and Drug Administration, PMD, FDA

## Abstract

On August 9, 2024, the CTIP symposium brought together various stakeholders in pediatric medical device (PMD) innovation to discuss the current state of pediatric medical devices (PMDs) and action steps that can collectively be taken to further drive PMD innovation. Meeting topics included 1) the Future of Pediatric Innovation, 2) Engaging Patients and Their Families in PMD Development, 3) Partnership Opportunities to Support PMD Research and Development (R&D), 4) Leveraging Real-World Evidence to Enhance PMDs, and 5) Fundraising and Investing in Pediatrics. This paper provides a comprehensive summary of the symposium proceedings, highlighting the critical needs, challenges, and opportunities in the PMD sector, and outlines potential areas for collaboration among stakeholders to drive progress in PMD development.

## Introduction

A great disparity lies between the commercialization of pediatric medical devices (PMDs) and adult medical devices. The number of PMDs approved is only a quarter of adult medical devices, with the majority of approvals being for pediatric populations ranging from 18–21 years old [1]. For the youngest and sickest patients, the gap is even wider. Out of 149 high-risk medical devices analyzed for age ranges of device premarket approval, the process by which Class III medical devices are assessed for safety and effectiveness, only 10 had a neonate indication and 32 had an infant indication [2]. This represents 28.2% of all devices approved for children. Currently, much of pediatric medical device (PMD) device use is off-label with little or no safety data in children, leading to inconsistent benefit-risk profiles from their intended use and a potential increased risk of negative health outcomes [3].

There are several limiting factors for PMD development for children, including the growth of children, differences in anatomy and physiology, and physical activity [4]. In addition, barriers to PMD commercialization include an often low return on investment due to a smaller market size, regulatory hurdles, special design constraints, and limited resources for research and development (R&D) [5]. To address the gaps in PMD development and translation, the Food and Drug Administration (FDA) created the Pediatric Device Consortia (PDC) Grants Program in 2009 after the passing of the Pediatric Medical Device Safety and Improvement Act of 2007 [6]. This program serves to provide funding and support for PMD development and to promote PMD accessibility. Over the past 15 years, across five grant cycles, 25 PDC awards have been received by 11 institutions, who in turn have assisted over a thousand PMD projects on their journey from concept to commercialization [4, 5].

## About The Consortium for Technology & Innovation in Pediatrics (CTIP)

CTIP is one of the five currently FDA-funded PDCs dedicated to the translation and commercialization of PMDs. Established in 2011, CTIP was first funded by the FDA in 2013, and its goal of improving child health outcomes by facilitating the development, production, and distribution of pediatric medical devices is manifested through its four-point mission statement:

1. **Accelerate** pediatric medical device development and commercialization through comprehensive wraparound services and non-dilutive funding at every stage of the total product life cycle.
2. **Connect** pediatric medical device innovators to our national network of experts and multidisciplinary stakeholders to foster research, clinical, and business partnerships.
3. **Advocate** for pediatric health equity across populations and diseases, and championing pediatric-specific innovation through research, publications, public events, education, and collaborations.
4. **Empower** founders, innovators, advocates, clinicians, and researchers from Underrepresented Minority (URM) backgrounds through meaningful allyship, active engagement and partnership, and deploying resources where they are needed the most.

**Fig. 1:**
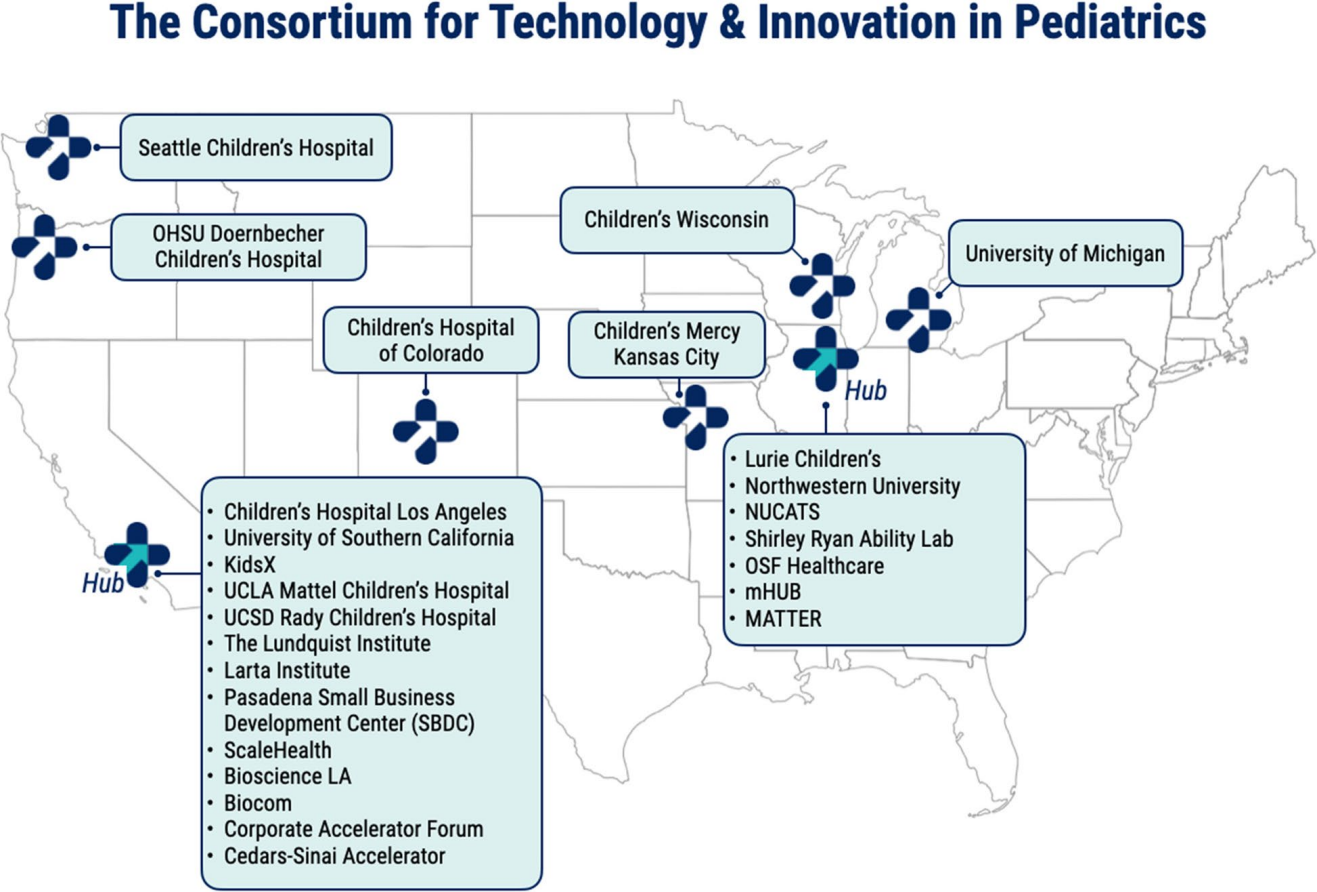
Location of CTIP 3.0 Network members

CTIP serves as a liaison between a variety of stakeholders in the evaluation and development of PMDs, promoting collaboration between patients and their families, device developers, regulators, clinicians, hospital administrators, investors, and industry. CTIP portfolio companies receive comprehensive assistance across the Total Product Lifecycle, which includes but is not limited to prototyping, IP protection, clinical trial design, regulatory strategy, and grant writing. Now in its third funding cycle, CTIP has experienced significant growth as it expanded from Southern California (2013–2018), to the West Coast (2018–2023), to the largest national network dedicated exclusively to the advancement of pediatric medical devices, representing 25 organizations across 8 states (Fig. 1) with hubs in Chicago and Los Angeles (2023–2028). CTIP has supported more than 220 device projects and helped bring 26 medical devices to market. The CTIP portfolio of companies has collectively raised over half a billion dollars in fundraising, including $40 million in non-dilutive federal grants.

CTIP hosts an annual Pediatric Device Innovation Symposium as an opportunity to bring together the MedTech community and focus on critical issues in pediatric medical device innovation (Table 1). The symposia serve as a catalyst to highlight successes, discuss challenges, nurture innovation, and create focused networking opportunities. Ann & Robert H Lurie Children’s Hospital of Chicago became the CTIP principal site in 2023, and so the inaugural symposium of the new grant cycle was hosted in Chicago for the first time on August 9th, 2024, at the Lurie Children’s and Northwestern University Medical Campus. Here, we present a summary of the topics discussed and insights shared in effort to share knowledge and resources that may be useful to pediatric device innovators who may not have been able to attend.

**Table 1:** CTIP symposium agenda for Friday August 9, 2024

Symposium Agenda
Welcome and Opening Remarks
Presentation: Pediatric Devices, CTIP, and the Future of Pediatric Innovation
Presentation: The Patient Voice: Engaging Children and Their Families in Medical Device Development
Panel 1: Engaging Patients and Their Families in Pediatric Medical Device Development
Portfolio Showcase 1
Panel 2: Partnership Opportunities to Support Pediatric Medical Device R&D
Panel 3: Real-World Evidence Opportunities to Advance Pediatric Medical Devices
Portfolio Showcase 2
Panel 4: Investing in Pediatrics
Closing Remarks

## Summit proceedings

### Presentation 1: Pediatric Devices, CTIP, and the Future of Pediatric Innovation with Dr. Juan Espinoza

#### Presentation Highlights

**Fig. 2:**
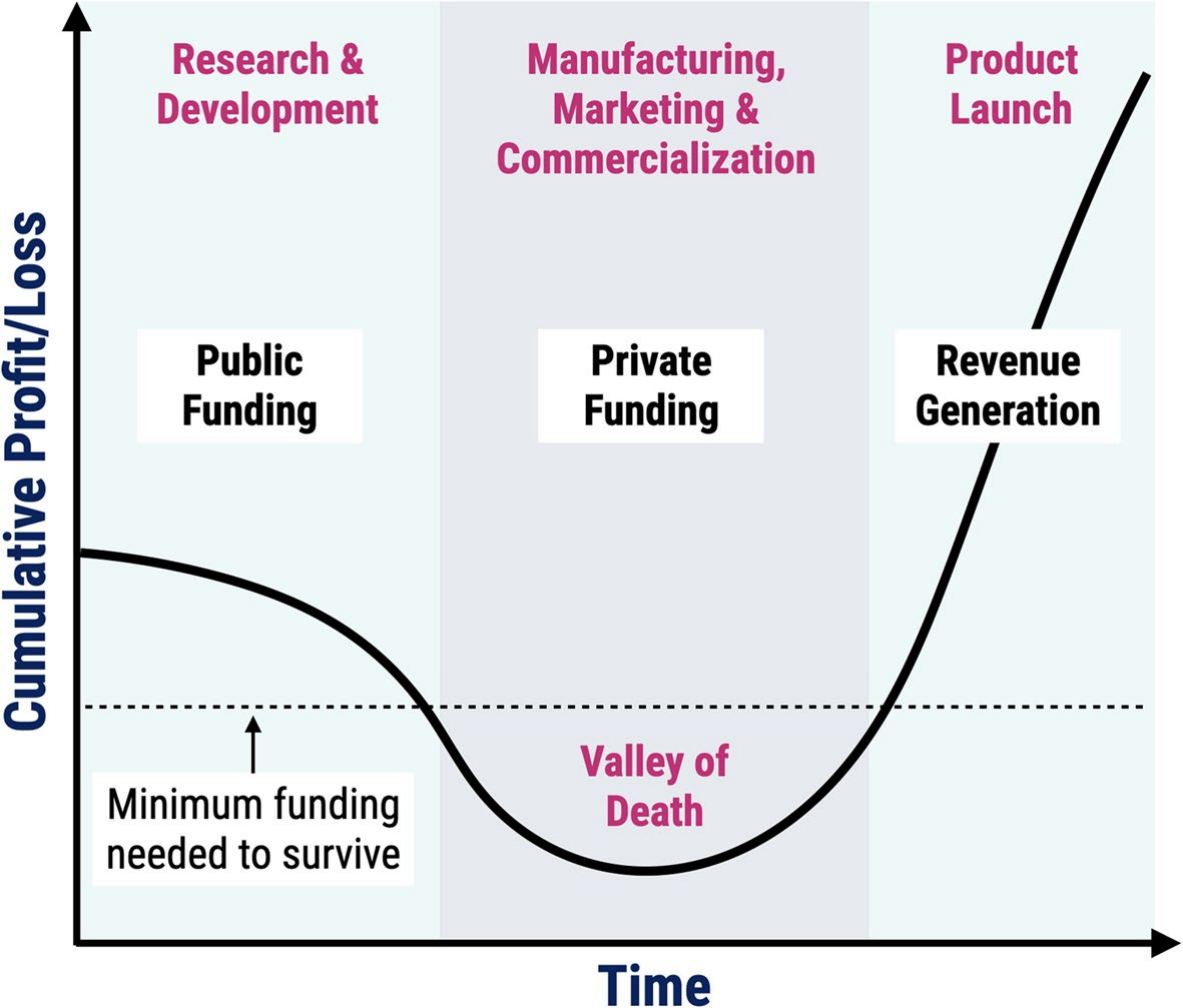
Illustration of the “valley of death,” occurring between R&D and the product launch

- Innovation for pediatric devices lags behind adult devices by as much as ten years.
- Financial challenges are the primary driver behind the lack of innovation and commercialization of PMDs.
- CTIP was developed to advance pediatric devices from concept to commercialization and to overcome the “valley of death (Fig. 2), " or the time where companies are operating without revenue.

To open the symposium, the first talk was given by Dr. Juan Espinoza, the principal investigator (PI) and Director of CTIP. Dr. Espinoza began his talk by giving an overview of the current state of PMD development, highlighting the various obstacles in developing PMDs. Several challenges were brought to the forefront: (1) the lack of safety data present due to the off-label use of medical devices for children, (2) the even larger disparities in device innovation for the youngest and sickest children, (3) the majority of work on device innovation being targeted towards adults, leading to medical device development being a decade behind for children compared to adults. Although there are numerous barriers hindering the translation of pediatric devices, including scientific and engineering, regulatory, and ethical challenges, the greatest highlighted were financial challenges.

When addressing the financial challenges, Dr. Espinoza outlined the disparity between the capital allocated to PMDs and the size of the pediatric population domestically. Pediatric patients comprise 25% of the U.S. population, but account for less than 10% of US healthcare dollars, 12% of research funded by the National Institutes of Health (NIH), and no more than 5% of health tech venture deals. When comparing similar healthcare services, reimbursement rates for pediatric care constitute only 50–70% of the adult coverage rates. Less than 10% of U.S. healthcare dollars are devoted to pediatric healthcare spending due to lower reimbursements for Medicaid compared to Medicare and lack of coverage for pediatric-specific treatments by the current payment model, indicating the need for policy change [7]. The origin of the disparity in PMD development lies in the steep costs and time it takes to develop pediatric medical technologies. The cost of medical device development ranges from $30 million to $200 million, while the timeline to get these devices to market is 3–10-years. Additionally, the pediatric markets have unstable revenue generation, which further prolongs the “valley of death,” or the time between concept and commercialization, where most inventions fail to move forward to commercial viability (Fig. 2). With these hurdles in mind, there is a great need to develop strategies that enable devices tailored specifically for children to survive the “valley of death” and make it to product launch and revenue generation. Such strategies may result in improved safety profiles due to fewer devices being used off-label and reduced healthcare disparities for children, ultimately promoting healthcare equity. There are several avenues from which this goal can be realized, ranging from regulatory and legislative actions to increasing research funding and industry support, ultimately shortening the time that innovations spend in the “valley of death.”

A number of organizations and initiatives are beginning to address the current limitations of PMD commercialization such as changes to Medicaid reimbursements, focusing on value-based agreements, and incentivizing industry partners to develop PMDs. The U.S. Congress attempted to address these challenges by implementing the PDC Grants Program to fund pediatric device consortia in 2007. Since the first funding cycle in 2009, the FDA has issued 20 awards to 10 institutions for a total of $14.6 M. By 2019, one thousand pediatric devices have been supported. CTIP was established in 2011 at Children’s Hospital Los Angeles (CHLA) and the University of Southern California (USC) with internal funding to facilitate collaborations to support PMD development. In 2023, CTIP expanded its presence to over two dozen organizations across multiple states, and was again funded by the FDA to continue its mission over the next 5 years. Thus far, CTIP has supported the commercialization of 21 marketable devices and supported portfolio members in achieving a totality of ~ 500 publications, $38.8 M of raised federal funding, and $422.7 M of raised venture capital funding. CTIP has held diversity and inclusion paramount in the portfolio selection process, and as a result, a significant percentage of companies are led by underrepresented founders, with 55% of founders identifying as female and 44% identifying as a person of color. At the end of his talk, Dr. Espinoza emphasized that through intentional action focused on bringing pediatric devices to market, we can change the shape of the “valley of death” curve to improve commercialization outcomes for these devices.

### Presentation 2: The Patient Voice: Engaging Children and Their Families in Medical Device Development with Dr. Carolyn Foster

#### Presentation Highlights

- Children with dependence and disabilities comprise 1–5% of the population but represent 50% of patient bed days
- Poorly designed medical devices have a negative impact on patient lives.
- Parents who have children dependent on medical devices often must develop creative solutions when their child’s medical device fails.

Dr. Espinoza’s talk was followed by an engaging lecture from Dr. Carolyn Foster, the Director of the Health@Home Initiative, which emphasized the importance of engaging the patient’s and their family’s voice in medical device development. As a physician who cares for children with medical technology dependence and disabilities, Dr. Foster pointed to this population as magnifying the problems that all children face when interacting with the healthcare system due to their frequency of interaction despite only making up 1–5% of the pediatric population. This population accounts for “50% of healthcare bed days,” or patients admitted to the hospital who require a bed [8], and “75% of intensive care unit resources [9].” She outlined the impact of design inadequacies on pediatric care, including malfunctioning of the devices, inability to customize the device to a growing child, insufficient durability, and inability to adapt the device to a home setting. These device challenges have a range of impacts, from affecting the quality of life of the patient and their family to potentially life-threatening consequences. By not including the patient's voice, simple but important aspects of device design are neglected such as optimizing ways to properly clean and transport devices. Given the increasing number of children being admitted to children’s hospitals over the last decade with an implanted device (> 30%) [10], it is critical for device developers to understand how devices can best function inside and outside of hospitals from the patient and their family’s perspective.

Dr. Foster raised another important consideration for medical device developers: Patients with complex medical care often depend on multiple devices. Therefore, when developing a medical device, it is essential to consider the various use cases and settings for which the device will be used. Patients and their families experience harms such as limited mobility, financial stress, poor health outcomes, and parental injury and stress due to poor device design. In addition to patient and family impacts, the health system also experiences the impacts of inadequate device design, including urgent evaluations due to device malfunctions, which comprise 30% of emergency room visits for this population, and extended hospital stays. Device abandonment is also a concern as insufficiently designed devices may go to waste. Reports from families indicated that poorly designed devices, especially monitoring devices, can both send patients to the hospital unnecessarily or miss important life-threatening symptoms. Due to these technology limitations, the families of device-dependent patients often resort to fixing life-sustaining medical devices on their own, using inexpensive but often temporary solutions. One example raised by Dr. Foster was a patient’s family using tape to seal a crack in a ventilator circuit. To address the gap between device designers and the patient voice, Dr. Foster facilitates interactions between patients, their caregivers, and device developers through her organization, Health@Home. Health@Homes’s mission is to transform healthcare delivery for pediatric populations using digital health technologies that can be deployed in a home or community setting [11]. Dr. Foster’s talk then segued into Panel 1 that further discussed how to engage patients and their caregivers in device development.

#### Panel 1: Engaging Patients and Their Families in PMD Development

**Moderator:**

Melissa Bent, MD.

Children’s Hospital Los Angeles, Los Angeles, CA, USA and Co-Director of CTIP.

**Panelists:**

Carolyn Foster, MD, MS.

- Ann & Robert H. Lurie Children’s Hospital, Chicago, IL, USA and Director of Health@Home Initiative

Melanie Turenne.

- Patient/Family Representative, Chicago, IL, USA

Nada Hanafi, MSC, MPH.

- MedTech Color, Encino, CA, USA

MicKayla Jones, MPH.

- Ann & Robert H. Lurie Children’s Hospital, Chicago, IL, USA

Leanne West.

- International Children’s Advisory Network (iCAN), Marietta, GA, USA

Panel 1 was designed and moderated by Dr. Melissa Bent, a co-Director of CTIP. She began the session with a powerful statement that, “Our goal…is to change the conversation of patient engagement from end-user to beginning partner.” The purpose of the panel was to demonstrate how patient involvement at the outset would add value to the device developer’s process. Dr. Bent started the panel discussion by asking Ms. Melanie Turenne about her experience as a mother who has a child with a complex medical condition and needs multiple medical devices to support his health and activities of daily living. Ms. Turenne’s son has Pelizaeus-Merzbacher Disease, a condition that requires constant monitoring and caregiving. This condition requires the use of a variety of medical devices not just for his survival, but for him to thrive and to be able to interact with his environment like other children. Dr. Bent then shifted the conversation to discuss the importance of medical device developers taking the feedback of the user seriously and making changes to their products based on that feedback. Ms. Turenne gave the example of the suction machine used by her son. The manufacturer made several changes that improved the ease of use such as rearranging the buttons to allow for charging while using the machine, and modifying the design to prevent secretions from being undesirably discharged from the machine. Although the main functionality of the device was unchanged, her experience as a user improved and made her feel that the company was listening to user feedback.

To give the audience some insights on how patients get involved in research that helps to inform device design, Dr. Bent asked Ms. MicKayla Jones, Manager of Research Operations at Smith Child Health Catalyst, how her and her team engages patients in research development and methodology. Ms. Jones collaborates with her team to assist researchers in engaging patients from the beginning of their research projects and helping them to think about how to work with patients at each step of their projects. Her work involves bridging the gap between researchers and patients, allowing them to feel included in the research process and desiring to persist with the research process so that the research brings about meaningful results and evidence-based changes. Ms. Nada Hanafi, Co-Founder & Board Director of MedTech Color, was asked to weigh in on this topic as well with the lens of how her organization engages patients from diverse backgrounds. Ms. Hanafi stressed the importance of streamlining this continuous feedback from patient advocates to device companies to account for individual patient needs. Similar to Ms. Jones’ team, Ms. Hanafi’s organization ensures that the patient’s perspective and patient-centered design thinking is prioritized from the initial stages of the design process to ensure adoption, compliance, and functionality of the device. Her organization includes patient advocates throughout the device lifecycle to understand the unique needs of the patients that are being served, the environments where the devices are being used, and parent interaction with the device.

The conversation then shifted to discussing how patient engagement resulted in improving a PMD. Ms. Leanne West, President of iCAN, mentioned how pediatric patients involved with her organization have provided insights for devices to make them more fun and engaging for children, such as adding gaming or including stickers. iCAN’s mission is to provide a forum for the pediatric patient and caregiver voice to inform innovation in healthcare, research, and clinical trials [12]. It is important to not only speak to parents but speak to the children as well to understand how to create a child-friendly device. As a physician who treats children with complex needs requiring medical devices, Dr. Carolyn Foster engages patients through patient advocacy and her own research, including patient-family advisors in every step of the research. One example she gave is her R21 grant regarding improving care for children with Downs Syndrome. In this grant, she intentionally included the patient-family voice where the co-PI on the grant had a child with Downs Syndrome, and also included a patient-family advisory board. Dr. Foster ended by encouraging researchers to not only have patients and their advocates involved in focus groups and surveys, but to imbed them in various levels of their study design.

Dr. Bent followed up by raising the importance of receiving iterative feedback as opposed to one-time feedback, and asked Ms. West to provide a framework for partnership survey design and suggestions for how to obtain feedback from the pediatric population. Ms. West’s organization iCAN serves as a bridge between PMD companies and the pediatric patients and their parents. Her organization assists companies in receiving feedback from patients and their parents by ensuring that the surveys are a reasonable length and reading level and making sure that the surveys address what is important to the children. After the companies make changes based on the children’s suggestions, ICAN shows the children how the surveys were changed in accordance with their input to demonstrate that their suggestions were taken seriously to encourage patient participation. Ms. West also encouraged the inclusion of patients in the study design so they can point out areas in the design (i.e. too many study visits) that may discourage their participation.

As a parent, Ms. Turenne shared similar sentiments to Ms. West that the amount of time a study will take is an important factor to patient and family participation. Ms. Turenne highlighted the need for transparency around what is expected from patient participation, such as the number of visits and what will be the primary method of communication. Flexibility surrounding study participation would encourage more patients and their families to participate. Incentivizing families through monetary compensation, allowing patients to keep the device, demonstrating genuine concern for patients, and communicating the changes made based on feedback from the patients and their families all go a long way in encouraging participation. Ms. Hanafi honed in on this idea of encouraging trust as alluded to by Ms. West and Ms. Turenne. She mentioned that it is essential to include diverse and representative voices in patient feedback given the historically poorer outcomes for women and minority racial and ethnic groups due to the lack of intentionality in including diverse perspectives. Exclusion of diverse voices often leads to a limitation in the understanding of the disease pathology in that particular group, restricting their access to effective preventative measures and treatments [13]. Lack of inclusion of historically marginalized populations, particularly in clinical trials, have also contributed to the image of medical research being exclusionary and therefore unworthy of trust [14]. From a socioeconomic lens, some patients may not have access to devices that they would need to participate such as a phone, computer, or internet access, leading to their exclusion. Proactively thinking about these barriers and ways to mitigate these barriers will encourage the inclusion of diverse voices and better outcomes for these populations.

Dr. Bent then asked the panel to elaborate on the challenges faced from a research and budgeting standpoint, particularly in the context of focus groups. One of the challenges faced by Ms. Jones’ organization is recruitment of focus groups on a constrained budget. Her organization addresses this by having those involved in community engagement review the questions to ensure that the questions asked are intentional if there are only a few focus groups that can be interviewed due to financial limitations. Her organization also prioritizes representation in the focus group so the device that is created is tailored to the intended end users to maximize the impact of the device in the absence of a large budget. In addition to budget constraints, Ms. West provided additional feedback on challenges to patient participation such as patients missing out on school or extracurricular activities due to research studies. She encourages researchers to see patients and families as people—considering their schedules, being cognizant about forming connections with them prior to asking them to participate in a study, and refraining from using language to refer to patients that may be dehumanizing such as “test subject.” To close the panel, Ms. Turenne brought out several points that she wanted device developers to remember when engaging patients and their families in device development (Table 2). To summarize the points, patients and their families are eager to provide feedback to device developers as they often face challenges with device usability and malfunctions. When this happens, they often have to innovate to keep their child alive, which may lead to fewer parents purchasing the device if their needs are not being met. Remember that the patient population is diverse, and the device may function differently when being used in the real world, so it is important to have iterative feedback.

**Table 2:** Panel 1 speaker insights

Speaker	Key Insights
Carolyn Foster	Include the patient voice at various levels of your study design, not just in receiving feedback through surveys or focus groups.
Melanie Turenne	No one is more passionate about providing you with feedback than a parent using a device to keep their children alive.
	Devices do not always work or are not tailored to patients and their family’s needs, causing them to use inexpensive and effective solutions to tailor devices to their needs. Allow patients and parents to inspire you.
	Patients and their caregivers have different abilities, backgrounds, and needs.
	Device design is missing the unpredictability of everyday life since it is often tested in a controlled environment. Patients and their caregivers can help device manufacturers to identify and uncover unmet needs, so it is important to include them from the beginning.
	Parents talk: To reach more people with a medical device, create a device that patients and their families love. Parents will tell other parents if they like or don’t like a device.
	Medical device companies should be flexible and openly communicate with parents regarding study requirements (number of visits, mode of communication). Monetarily compensating families for their time, allowing them to keep the device, and incorporating the family’s feedback into the device design may encourage trial participation.
Nada Hanafi	To meaningfully promote diversity and inclusion in clinical trials, it is important to remember that children are not small adults and to account for environment, parents, and use.
	Trust in clinical trials among historically marginalized ethnic groups can be facilitated by including diverse, representative, and inclusive people in the conversations, trial designs, and implementation.
	In addition to the need for culturally competent providers and researchers, it is important to consider the needs of the clinical trial participant that may hinder their ability to participate such as a phone and consistent internet access.
MicKayla Jones	To engage patients in clinical trials, patient engagement must be at the forefront for researchers from the beginning of the study.
	It is important to involve the community that is being studied in the study design. Have representatives of the community review the questionnaire.
Leanne West	Include feedback from pediatric patients in device and questionnaire development to ensure their concerns are addressed, not just parents.
	Showing the pediatric patients and their parents how you are incorporating their feedback into device development and your studies can incentivize future participation when requesting feedback.
	Remember that the patients and their families have obligations outside of research, so it is important to organize studies around their schedules and to limit the amount of follow-up visits required.
	Be mindful of the language used to refer to the patients on the consent forms. Calling the patients “test subjects” or “participants” may be perceived as dehumanizing.

#### Panel 2: Partnership Opportunities to Support PMD R&D

**Moderator:**

Seth Goldstein, MD, MPhil.

Ann & Robert H. Lurie Children’s Hospital, Chicago, IL, USA.

**Panelists:**

Emma Moran, PhD.

- CobiCure MedTech, New York, NY, USA

Steve Xu, MD.

- Sibel Health, Chicago, IL, USA

Katherine Maskel, MBA.

- Ann & Robert H. Lurie Children’s Hospital, Chicago, IL, USA

Nick Rydberg, MS.

- Minnesota Health Solutions, Minneapolis, MN, USA

Vasum Peiris, MD, MPH, FAAP, FACC, FASE.

- Center for Devices and Radiological Health (CDRH) at the Food and Drug Administration (FDA), Silver Spring, MD, USA

Panel 2 was designed by Dr. Grzegorz Zapotoczny, CTIP’s Medical Device Clinical Trials Unit Director and moderated by Dr. Seth Goldstein, a Pediatric General & Thoracic Surgeon at Ann & Robert H. Lurie Children’s Hospital of Chicago. The session started off with introductions from all of the panelists, where Dr. Emma Moran, Head of CobiCure MedTech, discussed how CobiCure supports PMD innovation through investment, collaboration, and most recently the creation of the CobiCure Fellowship for MedTech Innovation (Table 3). The CobiCure Fellowship was started in collaboration with the Pediatric Device Consortia, funding five fellows to work on developing PMDs in the areas of cardiovascular and critical care. The mission of this fellowship is to 1) create a portfolio of devices that meets the needs of pediatric patients, and 2) provide education and training for up-and-coming innovators in the distinctive aspects of pediatric device development.

**Table 3:** Panel 2 speaker insights

Speaker	Key Insights
Emma Moran	CobiCure, an organization designed to eliminate barriers to PMD commercialization, began a new fellowship in 2024 to foster innovation in PMD development and to train the next generation of PMD innovators.
	The PMD community is very supportive of each other due to a shared mission of getting PMDs into the hands of those who need them the most, so do not be afraid to ask for help.
Steve Xu	The best people to develop PMDs are mission driven.
	The different anatomies and physiologies of children as they grow from childhood to adulthood, make PMD innovation challenging.
	There is an urgent need to bridge the gap between clinicians and engineers to develop clinically relevant PMDs.
Katherine Maskel	Listen to people who are tangential to the problem you are trying to solve. Innovation can arise from cross-pollination between different problem solvers.
	Trust is key to effective communication and partnerships across sectors.
Nick Rydberg	Non-dilutive funding from the NIH such as Small Business Innovation Research (SBIR) funding can help to move clinical studies forward and get industry buy-in.
Vasum Peiris	The FDA has supported various initiatives such as the Pediatric Device Consortia Program and the Strategic Health Innovation for Pediatric-Medical Devices (SHIP-MD) program to advance development and innovation for PMDs, and advance market availability and patient access.
	These initiatives promote collaborative partnerships among public and private sector organizations that drive PMD innovation and health equity for children.

The 2024–2025 CobiCure Fellows are the following:

- James Reinhardt, PhD | Midwest Pediatric Device Consortium, Nationwide Children’s Hospital
- Jhalak Mehta, MS | Southwest-Midwest National Pediatric Device Innovation Consortium, Baylor College of Medicine and Texas Children’s Hospital
- Juliana Perl, MS | UCSF-Stanford Pediatric Device Consortium, Stanford University
- Stefano Pezzato, MD | Alliance for Pediatric Device Innovation, Children’s National Hospital
- Tamara Lambert, PhD | The Consortium for Technology & Innovation in Pediatrics, Ann & Robert H. Lurie Children’s Hospital of Chicago [15].

Following the discussion of CobiCure’s fellowship as a PMD support mechanism, Dr. Steve Xu, CEO of Sibel Health, offered his perspective on the unique design challenges that pediatric innovators face from a physician-engineer standpoint. During child growth and development from newborn to age 18, anatomies and physiologies vary greatly, illustrating that children are not “little adults” and require specific human design and adaptability considerations during PMD design. During her introduction, Ms. Katherine Maskel, Industry Engagement Manager at Ann & Robert H. Lurie Children's Hospital of Chicago, stressed the importance of building connections between hospitals and industry to foster innovation. Physicians have a deep knowledge of the hospital environment and unmet medical needs and can collaborate with industry on effective effort and resource allocation to bring pediatric solutions to market. Mr. Nick Rydberg, Vice President of Engineering at Minnesota HealthSolutions, discussed how his organization partners with MedTech entrepreneurs to leverage their engineering expertise. His organization assists with device prototyping and collaboration on NIH SBIR/STTR applications to secure funding for development, testing, and clinical studies to enable FDA regulatory submissions. Finally, Dr. Vasum Peiris, the FDA’s Chief Medical Officer and Associate Director for Pediatrics and Special Populations, discussed the FDA’s initiatives to facilitate pediatric device development. In addition to others, these initiatives include ongoing support for the PDC program and continuing development of the SHIP-MD program. SHIP-MD is a collaborative initiative initially proposed by CDRH’s Program for Pediatrics and Special Populations to create a national innovation ecosystem intended to mitigate the numerous challenges faced by pediatric medical device developers, and promote development of devices designed, evaluated, and labelled for pediatric populations.

Dr. Goldstein then asked the panel about ways collaboration and partnership can be facilitated. One of the benefits within the PMD stakeholders noted by Dr. Moran was a strong sense of community among the healthcare providers, patients, their families, the device developers, and entrepreneurs in this space. Given the large need for these devices, the PMD community is one where everyone generally wants to see each other succeed. As a result, Dr. Moran’s recommendation was to be open to new collaborations and partnership opportunities and to not be afraid to ask for help. Ms. Maskel’s approach is to continue to have events that bring problem solvers with different skill sets together to tackle complex issues with creative solutions. On a similar note, Mr. Rydberg underlined the importance of obtaining non-dilutive funding, such as NIH grants and recruiting clinical trial experts to de-risk the technology and make it attractive for industry collaborators and investors. Although many novel advances are occurring in the engineering field which may assist pediatric clinicians, Dr. Xu pointed out that there can be limited interaction between clinicians and engineers, resulting in a misalignment between the clinical needs and the engineered solutions. To alleviate this, meaningful collaborations in the PMD space need to start at the very beginning of the development. Ms. Maskel pointed out that the key element to building these meaningful collaborations is trust; particularly being able to communicate with each other effectively across various sectors despite the barriers between disciplines. With a national and global understanding, Dr. Peiris believes collaboration and partnerships can be facilitated by clarifying the interconnection of organizational values, and synergistically engaging the comparative strengths of partners toward common goals that serve the collaborators, the collaboration, and the ecosystem. In this case, an ecosystem that serves the medical device needs of children and their families.

Diving into the question of public–private partnerships, Dr. Peiris highlighted the national initiative, SHIP-MD which has received tremendous support from public and private sector partners including the FNIH, NIH, BARDA, CobiCure, AdvaMed, the American Academy of Pediatrics, multiple pediatric hospitals, and others to drive pediatric device innovation. The Critical Path Institute (C-Path) served as the neutral third-party convenor for the first phase of SHIP-MD development, and the Foundation for the National Institutes of Health (FNIH) is serving as the current third-party convenor for the initiative. Given that in some specialties, up to ~ 90% of devices being used for children are being used off label and the benefit-risk profile for these devices have not been submitted for evaluation by the FDA, there is a need to create partnerships like those enabled by SHIP-MD to foster an equitable health landscape for children. Dr. Xu stressed the importance of having infrastructure in place to support devices that are specifically designed for kids but also devices that can be used by patients of all ages without an unfavorable benefit-risk profile for kids. To conclude, the panelists shared their advice to innovators on the subject of partnerships and collaborations. Ms. Maskel encouraged innovators “*not to overthink the process*.” Partnerships and collaborations do not have to be determined at the very beginning, but it is important to think about them early to brainstorm how to bring a safe and effective medical device to market. Dr. Peiris noted the availability of the FDA-supported PDCs, such as CTIP, intended to support PMD innovators with guidance and support throughout the device’s life cycle.

#### Panel 3: Real-World Evidence Opportunities to Advance PMDs

Moderator:

Juan Espinoza, MD.

Ann & Robert H. Lurie Children’s Hospital, Chicago, IL, USA.

Panelists:

Charles Viviano, MD, PhD.

- Office of Clinical Evidence and Analysis (OCEA), Center for Devices and Radiological Health (CDRH), Food and Drug Administration (FDA), Silver Spring, MD, USA

Cori Maegley, M.S., MBA.

- National Evaluation System for health Technology (NEST), an initiative of Medical Device Innovation Consortium (MDIC), Arlington, VA, USA

Katie Mues, PhD, MPH.

- Aetion, New York, NY, USA

Stacey Ellul, RN, MBA.

- NeuroPace, Mountain View, CA, USA

Panel 3 was designed and moderated by Dr. Juan Espinoza, the principal investigator (PI) of CTIP. The panel was first asked to define real-world data and real-world evidence, and its role in medical device regulation. Dr. Charles Viviano, Chief Medical Officer at OCEA, defined real-world data from the FDA’s standpoint as “data relating to patient health status and/or the delivery of health care routinely collected from a variety of sources.” CDRH reviews this data to support regulatory objectives. As part of its evaluation of RWD, the FDA assesses the relevance and reliability of the data. Data from real-world and experimental sources are both subjected to the same rigorous evidentiary bar and scrutiny (Table 4). When asked about the necessity of real-world evidence for PMD innovation from a company’s perspective, Ms. Stacey Ellul, Vice President, Quality & Regulatory, NeuroPace, emphatically stated the importance of this evidence and the need for collaboration with the FDA when developing clinical device studies for pediatric patients.

**Table 4:** Panel 3 speaker insights

Speaker	Key Insights
Charles Viviano	The FDA holds evidence from clinical trials and real-world evidence to the same evidentiary bar.
	Leveraging existing data routinely collected during the use of PMDs represents a tremendous opportunity to capture real-world data and support the objectives of a real-world evidence study.
Cori Maegley	NEST functions as a convener, bringing together industry and regulatory collaborators to assist device manufacturers in generating real-world evidence suitable for use in regulatory submissions.
	These collaborations may lead to successful label expansion, reducing the need to use devices off-label.
Katie Mues	Involve regulators in your design process. Request feedback regarding how often the clinical outcome is being measured in the study versus the real world as this may impact your results.
	Take time to understand the potential databases you may use for your real-world study and get feedback from stakeholders before determining which one(s) to use.
Stacey Ellul	Allow the data to drive decision making and device development, not passion.
	Collaboration between device companies and clinical investigators are essential to prevent off-label device use in children.

Ms. Ellul described her company’s journey to bring their active implantable neurostimulator device to treat drug resistant focal epilepsy to market. NeuroPace started their original pivotal study in 2005; they enrolled 240 patients and implanted 191 with their device in a double-blind, sham controlled study. It took three years to enroll the patients, and eight years before receiving FDA approval in 2013. The indication at the time was for patients 18 years and older. While going through this process, NeuroPace continued to collect long term data, and added new patients during an open label period, many of whom were 18 to 21 years. In 2013, NeuroPace presented data to the FDA of 50 patients ages 18 to 21 years but was not able to come to an agreement with the FDA on a plan to expand pediatric labeling through extrapolation of the data.

In 2019, NeuroPace performed a retrospective chart review with an additional 20 patients, again with the goal of obtaining a pediatric labeling expansion for 12—18 years, but this too was unsuccessful. NeuroPace commenced a pediatric specific pivotal study with a goal of enrolling 200 patients a few months before COVID reduced Comprehensive Epilepsy Center treatment activities, and struggled to recruit since. Ms. Ellul reflected that it has been almost 20 years since NeuroPace started clinical trials, and they still have not been able to generate the pre-market data required to obtain a pediatric indication. This has the very real downside that patients who desperately need access to this technology cannot receive it. This realization led NeuroPace to work collaboratively with the FDA to consider using real world data from current practice of medicine experience, (often referred to as ‘off-label’ use) to demonstrate safety and efficacy in a pediatric population. There was a brief discussion about the importance of “not going alone” on a regulatory journey, particularly when considering the use of real-world data to support a marketing application.

Dr. Espinoza then directed the discussion to Ms. Cori Maegley, Vice President of Partnerships Management at NEST, asking her to describe the role NEST plays to ensure that companies do not have to go on the real-world data journey alone. NEST is a public–private partnership funded by Medical Device User Fee Amendments (MDUFA) fees with the mission of catalyzing the use of real-world evidence (RWE) in the medical device ecosystem. NEST acts as a convener between industry and the FDA to address some of the challenges faced by medical device companies when submitting regulatory documents to the FDA using real-world data. NEST addresses these challenges by finding high-quality and reliable real-world data sources, and making sure there are available processes and mechanisms to advance appropriate use of real-world data in regulatory submissions. This helps to ensure that the submissions withstand the regulatory framework of the FDA, and provides assurance for medical device companies that using real-world evidence is not riskier compared to a traditional clinical trial. Through these processes and frameworks, NEST is helping to standardize the documentation that the FDA receives from medical device companies, which helps to create a consistent and reliable experience for both the FDA and medical device companies.

In expansion of this discussion, Dr. Espinoza asked Dr. Katie Mues, Vice President of Science & Delivery at Aetion, how one should properly source real-world data that leads to real-world evidence from her and her company’s perspective. Aetion encourages clients to design their study as a target clinical trial and then conduct the real-world evidence study with this framework. Afterward, those designing the study should look for a dataset that meets the minimal set of variables or includes required variables and optionally, nice-to-have variables to ensure that the study can meet its primary endpoint. It is important to speak with regulators to determine if there are any disparities in the frequency in which the clinical outcome is being measured in the study versus the real world and how that may impact study results. It is also imperative to have a deep understanding of all of the potential databases you may use for your study and get buy-in from all partners before making your selection. Aetion has a partnership with NEST, helping to bring these conversations to the forefront for innovators to consider while designing their studies. Ms. Ellul reiterated the point brought up during the previous panel about the importance of patient-centricity and feedback in device development. It was shared that it is common for pediatric patients and their caregivers to be open to accepting a different benefit-risk profile than the one deemed acceptable for an adult patient due to the burden placed on the families and the desire for normalcy. Centering the patient in these discussions is essential to being mission-driven because the burden and benefit-risk calculations are different for a pediatric patient expected to live with their health condition for several decades versus an elderly patient who may not have the same life expectancy.

The conversation shifted to discuss the rationale and opportunities to collect real-world evidence, including when; 1) conducting randomized trials may be unethical or infeasible, 2) there is a high unmet medical need, 3) there is a deep understanding of the efficaciousness of the intervention (device, therapy, or vaccine), and 4) there is significant off-label use to support a study, either in the US or another jurisdiction. Dr. Mues pointed out that these opportunities exist in the pediatric medical device space unlike many other clinical spaces. In closing, panelists discussed what they are most excited about in regard to real-world evidence and PMDs. Dr. Viviano believed leveraging existing data routinely collected during the use of PMDs represents a tremendous opportunity to capture real-world data and support the objectives of a real-world evidence study. Ms. Maegley’s perspective was that off-label use often exposes patients to an unknown risk–benefit profile and places companies in the tenuous position of trying to support clinical users of their devices with limited data and limited ability to provide direct clinical support and education. Given these risks, she demonstrated excitement about collaborators being brought together to change the current situation surrounding off-label use. Dr. Mues concurred that she is also excited about the partnership and the willingness of each party in the ecosystem to build together. Ms. Ellul provided closing thoughts, admonishing developers and researchers that the shape of the “valley of death” curve can be changed if they allow the data to guide them.

#### Panel 4: Investing in Pediatrics

Moderator:

Madison Christmas.

Ann & Robert H. Lurie Children’s Hospital, Chicago, IL, USA.

Panelists:

Andrew Meadow, MBA.

- Health Innovation Capital, Chicago, IL, USA

Kathryne Cooper, MBA.

- The Consortium for Technology & Innovation in Pediatrics, Los Angeles, CA, USA

Alexandra Shandiz, MBA, MS, OTR/L.

- Kaiser Permanente Ventures, Oakland, CA, USA

David Kereiakes, MBA.

- Windham Capital Partners, New York, NY, USA

Dana Sun, MBA.

- Laerdal Million Lives Fund, San Francisco, CA, USA

The last panel was designed and moderated by Ms. Madison Christmas, CTIP’s Program Associate of Investment and Growth. Ms. Christmas started with the statistic that novel pediatric medical devices number only a quarter of those designed, evaluated, and approved for those of adults. The few devices that are designed or intended for children typically only address the needs of individuals 18 years and older. Considering the vast opportunity for pediatric device development, she asked the panel about emerging trends in the pediatric market that investors should look forward to. Mr. Andrew Meadow, Founding Partner and Chief Investment Officer at Health Innovation Capital, believes that we are at a crossroads in PMD innovation due to the historically limited development of PMDs until 2007. This is when changes in the regulatory process impacted reimbursement, allowing for more PMDs to make it to market. Although investors commonly believe that investing in pediatrics will not yield a substantive return, Mr. Meadow demonstrated that the trend in reimbursement for pediatric products has increased due to the changes in the pediatric regulatory and payor environment over the last seven years. Payment for drugs for diseases like cystic fibrosis and sickle cell anemia that affect children has been approved in recent years, setting a standard for how much payors are willing to reimburse for similar treatments in the future. He also mentioned the incentives available to motivate companies to develop products for the pediatric space, such as the voucher program, orphan disease designation, and the compassionate use designation. These changes in the regulatory and payor landscape have paved the way for pediatric medical innovations to make it to market, but unfortunately, many still do not make it. Mr. Meadow’s perspective is that oftentimes, a greater contributor to this lack of commercial success is a misalignment between company vision and supportive partners, rather than financial resources. Access to capital is not as great of a contributor to the “valley of death” as is making sure companies and their vision are matched with the right partners and audience.

Ms. Christmas then dove deeper into the barriers that reimbursement and market size pose on PMD developers with respect to venture capital investment. The panel was asked to address what prevents early-stage investors from investing in PMDs and how founders can address these concerns proactively. Ms. Alexandra Shandiz, Senior Associate at Kaiser Permanente Ventures, like Mr. Meadow, contributed to the narrative that there is recent interest in investing in PMDs due to changes in the regulatory environment. However, one barrier to investment for early-stage investors is extensibility, or the ability of the product to translate into use in the adult population to ensure a substantial return on investment. For mid-to-late-stage investors, the barrier to investment is commercial traction. There are a variety of funds to assist in PMD development besides investment funds, including corporate funds and institutional-based funds. Nevertheless, what is most essential for PMD developers is finding the right partner that understands the benchmarks they need to meet to achieve success. Ms. Kathryne Cooper, CTIP’s Investment Advisor, Emeritus, further elaborated on how PMD developers can de-risk their technologies by leveraging non-dilutive and public funding opportunities. She noted how CTIP specifically provides non-dilutive grants to PMD companies through funding provided by the FDA (Table 5). Healthcare companies can also take advantage of other government funding opportunities such as SBIR, STTR, NSF grants, and AFOSR grants. CTIP helps connect PMD innovators with grant application support resources, such as grant writers and has achieved marked success in helping their innovators obtain funding. Government funding allows PMD developers to achieve the milestones required for external investors without sacrificing equity.

**Table 5:** Panel 4 speaker insights

Speaker	Key Insights
Andrew Meadow	Changes to the regulatory and payor landscape have assisted in lowering the barrier to PMDs entering the market.
	Lack of effective partnerships is a greater contributor to the failure of a PMD company than finances.
	Ensure that the data works for developed and developing markets to prevent the worsening of health inequalities.
Kathryne Cooper	Non-dilutive government funding can assist in achieving required benchmarks and de-risking PMDs for investors.
	CTIP assists PMD innovators by connecting them with resources to help them obtain both dilutive and non-dilutive funding.
Alexandra Shandiz	Early-stage investors look for PMDs that can be translated into the adult medical device market to improve the return on investment.
	Mid to late-stage investors seek commercial traction when evaluating investment opportunities.
	PMD companies need to find the right partners to help them meet the appropriate benchmarks for success.
David Kereiakes	Be transparent with investors regarding your market size to find the right partners.
	Collect data as soon as possible to maximize economic and clinical benefit.
Dana Sun	PMDs will not be commercially successful without the appropriate evidence, reimbursement strategy, and advocacy by key opinion leaders (KOL).
	PMD companies should focus on collecting high-quality, longitudinal data with predictive potential.

Even when a company surpasses the valley of death by reaching a product launch, there are still obstacles that they need to overcome, such as sales, reimbursement, and continuous customer discovery. To this point, Ms. Christmas asked the panel to elaborate on how companies can build strong sales teams and go-to-market strategies. Mr. David Kereiakes, Managing Partner at Windham Capital Partners, spoke about his experience in leading an investment in a start-up company. His organization sold and licensed the rights of the company’s technology to a larger company to invest non-dilutive funding in the sales and marketing and R&D arms of the company. He also emphasized the importance of being transparent regarding the size of the target market, even if it is a small market. This will help to attract the right investors. Ms. Dana Sun, Principal at Laerdal Million Lives Fund, brought to the forefront two major impediments for medical device companies: 1) generating the necessary evidence to persuade payors and 2) generating the appropriate KOL advocacy in guidelines to clinicians to educate them on why the device should be used. Even if PMD device developers receive FDA clearance, without the necessary evidence, reimbursement strategy, and KOL advocacy, the device will not be commercially viable. She strongly recommended incorporating planning for these key elements much earlier in the device development lifecycle as PMD innovators are developing their regulatory strategy.

In the Q&A session, an audience member asked about the role that the underlying data plays in investors determining whether they should invest in a company. In Mr. Meadow's estimation, investors see data as currency. Investors want to know about the quality and integrity of the data, who would potentially want to access the data, and if it can be compiled meaningfully for stakeholders. It is important to demonstrate that the data not only works for developed markets, but developing markets as well to ensure that healthcare inequality is not further exacerbated. Mr. Kereiakes brought out the importance of timing, to ensure that the data is collected as soon as possible to maximize the clinical and economic benefit. Ms. Sun suggested not to just concentrate on the volume of data, but to collect high quality data that is longitudinal and has predictive potential. Ms. Shandiz provided the final thought, expressing that PMD developers need to disclose to health systems what they are doing with the data, how they will protect it, and only to request data that they need.

### Symposium Showcase Highlights

CTIP highlighted several portfolio companies in the CTIP Symposium showcase, done by Ms. Bianca Riello, CTIP’s Program Associate of Networking and Partnerships. The companies highlighted their innovative technologies that are set to revolutionize the current state of pediatric care. The companies that presented and a highlight of their presentations are below:

- *Rhaeos* has developed a non-invasive, wireless, wearable device that assesses shunt flow in minutes, reducing the length of hospital stays, readmission costs, and unnecessary imaging. “It is not a matter of if (a shunt fails), it is really a matter of when… It is not uncommon for kids to have more shunt surgeries unfortunately rather than birthdays.”
- *Atrillity Medical* has developed a continuous atrial electrogram to identify post-operative cardiac arrhythmias, improving time to diagnosis and treatment. “Pediatrics and babies have a really fast rhythm with signals not as strong as adults… It’s hard for a clinician to see their rhythm… as clearly as they might need to. Depending on what is seen or not seen on that monitor, a clinician might lead to a different diagnosis or course of action which might make a big difference in that baby’s life.”
- *Annoviant* is developing a regenerative heart valve for heart disease capable of healing and growing with the patient, preventing the need for multiple procedures. “The technology has not changed in the last 60 years… The devices that are currently used degenerate much faster in pediatric populations.” Annoviant mentioned in a follow-up discussion that their preclinical data suggests they can make their device functional and durable with lower complications.
- *Cast21* has developed a twenty-first century waterproof cast alternative replacing traditional cast technology, allowing for a clean, easy, and quick way to set broken bones. “At Cast21, we focus on elevating the patient experience and decreasing medical frustration starting with orthopedics.”
- *Happiest Baby* has developed the SNOO, a smart sleeper that soothes fussing babies back to sleep in under a minute and keeps babies lying securely on their back to lower the risk of SIDS. “Parent exhaustion can lead to postpartum depression, child abuse, and ultimately unsafe sleeping practices that leads to thousands and thousands of deaths per year. What if we could reduce infant death, and get people back to work? What if we could make parents feel happier and supported even if they don’t have an extended family?”.
- *Remmie* is developing an otoscope that enables physician detection of ear infections from a remote care setting, preventing unnecessary patient doctor’s visits. “Today, a diagnosis that’s unsupported by Remmie.ai is 30% less accurate than a diagnosis supported by Remmie.ai. This leads to better outcomes for patients, providers, and payors at large.”

### Symposium Reach and Impact

As seen in Table 6, 313 people registered for the Symposium. Of those, 200 attended the event; 96 in-person and 104 virtually. People from 25 states and 9 countries registered for the Symposium; Illinois, California, and Texas had the most registrants. Most individuals (~60%) heard about the Symposium from either CTIP’s social media and newsletters, or from colleagues or friends, highlighting the importance of active networking and communication in the MedTech industry.

**Table 6:** Characteristics of 2024 symposium registrants and attendees

Characteristics	Registrants (*n* = 313)	Attendees (*n* = 200)
**Industry Sector**		
Academia	93 (30%)	54 (27%)
Entrepreneur or Startup	80 (26%)	56 (28%)
Research	89 (28%)	48 (24%)
Healthcare Provider	71 (23%)	38 (19%)
Innovation Services	55 (18%)	36 (18%)
Industry	27 (9%)	21 (11%)
Investors and Philanthropy	25 (8%)	14 (7%)
Government and Regulatory	12 (4%)	9 (5%)
Patient or Patient Advocate	12 (4%)	8 (4%)
Other or No Data	52 (17%)	39 (20%)
**Location**		
Midwest	160 (51%)	93 (47%)
West	46 (15%)	33 (17%)
South	23 (7%)	17 (9%)
Northeast	17 (5%)	11 (6%)
Mid-Atlantic	16 (5%)	11 (6%)
International	14 (4%)	6 (3%)
No Data	37 (12%)	29 (15%)
**Source of Awareness**		
CTIP Media Channels	105 (34%)	72 (36%)
A Colleague or Friend	87 (28%)	50 (25%)
Hospital Media Channels	41 (13%)	19 (10%)
Other	60 (19%)	37 (19%)
No Data	55 (18%)	46 (23%)

The symposium featured an engaging Q&A session where attendees asked thoughtful questions about pediatric device innovation. A key topic was the collaboration between hospitals and startups. Many questions focused on how hospitals can work better with innovators, especially in understanding critical processes like procurement and purchasing, hospital billing, and value analysis committees. For instance, one participant asked about best practices for hospitals to build stronger partnerships, highlighting the need for clear communication and structured engagement.

Another important discussion point was the cost and planning of feasibility studies for clinical validation in pediatric hospitals. Participants were concerned about the financial challenges startups face and called for more transparency and support. Trust in strategic relationships was also emphasized, with questions about managing risks and resolving conflicts between startups and hospitals. These discussions underscored the complex challenges of integrating new solutions into pediatric healthcare and the importance of creating collaborative environments.

After the event, 40 individuals (20% of attendees) completed online evaluation forms about the Symposium (17 from online participants and 23 from in-person participants). The evaluation consisted primarily of 5-point Likert scale items, single select items, open-ended questions, and a net promoter score item on a scale from 0–10; Table 7 summarizes relevant responses. Feedback was overall positive, indicating a high level of satisfaction with both the content and the format, as well as a high likelihood of attending future events and recommending CTIP events to others.

**Table 7:** Participant Feedback (*n* = 40)

**Panel Feedback**	
Relevance	4.53
Duration	4.55
Knowledgeable Speakers	4.83
Applicable information	4.53
**Event Length**	
Just Right	85%
Too Short	13%
Too Long	2%
**Event Composition**	
Balanced	74%
More Talks and Panels	21%
More Networking	5%
**Overall Feedback**	
Satisfied with Event	4.73
Likelihood of Joining in 2025	4.6
Net Promoter Score	9.45

In the open-ended questions, respondents mentioned the quality of the speakers (particularly the presence of FDA representatives); the balance of talks, panels, and networking time; actionable regulatory insights; and the opportunity for both in person and hybrid participation as highlights of the event. Opportunities for improvement included better communication of day-of logistics, more time for asking questions of panelists and experts, and more industry representation. Attendees also offered several suggestions for future topics, expressing interest in exploring go-to-market strategies, commercialization pathways, and methods and strategies to move from grant-supported research to market-ready pediatric devices. Among those that identified as entrepreneurs. industry, or vendors, half expressed interest in being sponsors or exhibitors at next year’s symposium.

## Conclusion

The 2024 CTIP Pediatric Device Innovation Symposium presented a comprehensive overview of the field and covered key aspects of PMD commercialization, including patient engagement, the patient voice, partnership opportunities, real-world evidence, investment strategies, and future trends. The primary goal was to identify key factors that affect PMD commercialization and to unify stakeholders around the mission of advancing PMD commercialization through partnership, collaboration, and knowledge sharing. The ultimate aim of these efforts is to increase the likelihood that PMDs will overcome the challenges discussed and reach the pediatric patients who need them most.

## Data Availability

The link to the original recordings is the following: https://www.youtube.com/playlist?list=PLmYcGUbASqk8_euEFumD40NJe0ou9GhgK

## Abbreviations

AFOSR: Air Force Office of Scientific Research
CDRH: Center for Devices and Radiological Health
CHLA: Children’s Hospital Los Angeles
C-Path: Critical Path Institute
CTIP: The Consortium for Technology & Innovation in Pediatrics
FDA: Food and Drug Administration
FNIH: Foundation for the National Institutes of Health
iCAN: International Children’s Advisory Network
KOL: Key opinion leader
MDIC: Medical Device Innovation Consortium
MDUFA: Medical Device User Fee Amendments
NEST: National Evaluation System for health Technology
NIH: National Institutes of Health
NSF: National Science Foundation
OCEA: Office of Clinical Evidence and Analysis
PDC: Pediatric Device Consortia
PMD: Pediatric medical device
PI: Principal investigator
R&D: Research and development
RWE: Real-world evidence
SBIR: Small Business Innovation Research
SHIP-MD: Strategic Health Innovation for Pediatric-Medical Devices
STTR: Small Business Technology Transfer
USC: University of Southern California
